# Vibration Band Gap Characteristics of Two-Dimensional Periodic Double-Wall Grillages

**DOI:** 10.3390/ma14237174

**Published:** 2021-11-25

**Authors:** Chuanlong Wang, Xiongliang Yao, Guoxun Wu, Li Tang

**Affiliations:** College of Shipbuilding Engineering, Harbin Engineering University, Harbin 150001, China; wangchuanlong@hrbeu.edu.cn (C.W.); xiongliangyao@hrbeu.edu.cn (X.Y.); tanglitly@hrbeu.edu.cn (L.T.)

**Keywords:** wave finite element method, double-wall grillage, band gap characteristic, frequency transfer function

## Abstract

In this article, the wave finite element method (WFEM) is used to calculate the band gap characteristics of two-dimensional (2D) periodic double-wall grillages (DwGs), which are verified by the grillage model vibration measurement experiment and finite element calculation. To obtain the band gap characteristics of periodic DwGs, the finite element calculation model is established according to the lattice and energy band theory and the characteristic equation of the periodic unit cell under the given wave vector condition is solved based on Bloch theorem. Then, the frequency transfer functions of finite-length manufactured and finite element models are obtained to verify the band gap characteristics of periodic DwGs. Finally, the effects of material parameters and structural forms on band gap characteristics and transfer functions are analyzed, which can provide a reference for engineering structure vibration and noise reduction design.

## 1. Introduction

Grillages have been commonly used in various vehicles and engineering structures, such as aircraft, ships, and bridges. As the basic component, its dynamic characteristics directly determine the vibration response of overall structures, which in turn affects the working accuracy of equipment and the service life of the structure. Therefore, it is of great significance to study the vibration characteristics of grillage structures. So far, the research on dynamic characteristics of grillage structures has mainly focused on single-wall grillages (SwGs) or the orthogonal stiffened plate. In 1956, Hoppmann et al. [1] converted a square stiffened plate into an anisotropic plate to calculate the natural frequency and verified it through experiment. Subsequently, Iyengar et al. [2] derived the characteristic equation of stiffeners and solved the problem. With the development of computer technology, the finite element method (FEM) has become the main force of vibration and buckling analysis of stiffened plates [3,4]. However, compared with SwGs, DwGs have higher strength and stronger vibration attenuation capability but have not attracted more attention [5]. Only a few scholars have studied their sound transmission characteristics as a sound insulation material [6,7,8]. In recent years, the vibration band gap characteristics of periodic structures have become a research hotspot. Especially in large-scale structures composed of DwGs, the existence of periodicity will produce a frequency-response-forbidden band in which the elastic wave will rapidly decline so as to suppress the vibration transmission in the grillage structures. This will be conducive to controlling the vibration transmission through structural design and inject new vitality into the study of dynamic characteristics of grillage structures. At the same time, the study found that periodic structures not only have the band gap characteristics of suppressing vibration transmission but also demonstrate better performance in terms of crack arrest and fracture resistance in some elastic wave metamaterials [9], which can be proved by formula derivation of the energy release ratio and numerical results. The discovery of this new phenomenon will help improve the structural strength and the vibration isolation performance of periodic structures and metamaterials with local resonators, endowing them with broader application prospects.

Looking back on the development of vibration analysis of periodic structures, Brillouin et al. [10] pointed out as early as in 1953 that there were energy band gaps in periodic structures that hinder the propagation of elastic waves, and in the 1970s, Mead et al. [11,12,13] introduced the wave method into the calculation of vibration characteristics, which greatly promoted the study of periodic structures. In 1993, Kushwaha et al. [14] first proposed the concept of phononic crystal, and then Liu et al. [15] proposed the theory of the local resonance band gap, which further expanded the application of band gap characteristics in low-frequency vibration. In the progress of band gap theory, numerical methods for different structures emerge endlessly. The vibration characteristics of 1D beams or layered structures are usually solved by the transfer matrix method or the reverberation-ray matrix method [16,17]. The plane wave expansion method and its improvement method are usually adopted to calculate the band gap characteristics of regular-shaped structures to ensure the convergence of results, such as 2D plate structures [18,19,20]. In addition, there are multiple scattering methods for cylindrical or spherical scatterers [21,22], the finite-difference time-domain method for finite size phononic crystals [23,24], etc. In recent years, the spectral element method has been gradually applied to the solution of the vibration band gap of periodic structures with high accuracy and calculation efficiency and satisfactory results have been obtained [25,26]. Since more complex physical models cannot be solved due to the limitations of algorithms and FEM has been widely used in various fields of scientific research and engineering applications [27,28], the method on the basis of FEM combined with periodic boundary conditions to calculate the complex structure vibration band gap provides effective assistance in solving such problems [29,30,31], which also means that the WFEM used in this paper has a broad application prospect.

As a general numerical method, the WFEM is suitable for most types of structures and materials with good convergence, and therefore a series of scholars have applied the theory to solve problems, for example, related to bars, beams, plates, or other complex 3D structures in recent years. Nobrega et al. [32] obtained wave band gaps in an elastic metamaterial rod by the WFEM, which were verified via the spectral element method. Wen et al. [33] gained flexural wave transmissions of periodic beam grid structures and verified these through experiment. Mace et al. [34] calculated dispersion relations of the thin plate, the asymmetric laminated plate, and the laminated foam-cored sandwich panel. El-Sabbagh et al. [35] optimized the topology of Mindlin plates based on the WFEM to achieve the best natural frequency and band gap width. Li et al. [36] studied the propagation characteristics of Lamb waves on a 1D radial phononic crystal plate with periodic corrugations and discussed the effects of geometric parameters on band gaps. Liu et al. [37] obtained the band structure of 2D square lattices using plane elastic elements based on a B-spline wavelet on the interval and compared it with traditional FEM, which provided good results. Xiang et al. [38] proposed a 2D surround multi-scattering phononic crystal structure and calculated the band gap and transmission characteristics, which is helpful in the research and design of acoustic functional materials. Warmuth et al. [39] studied the band gap characteristics of a novel cellular material consisting of only one phase, while Meng et al. [40] found that a 3D lightweight rainbow structure has ultra-wide band gaps. Matlack et al. [41] and D’Alessandro et al. [42], respectively, proposed a 3D periodic structure achieving low-frequency and wide-bandwidth vibration band gaps, which were verified through product manufacture and experiment. To sum up, the WFEM can be applied to most aspects of vibration band gap analysis, which plays an important role in the theoretical research on and practical application of periodic structures.

The research work mentioned above mainly focused on simple periodic structures or newly designed periodic structures, while there are usually a large number of periodic grillage structures in complex structural systems, such as ships and aircraft. A full study of vibration band gap characteristics is conducive to guiding the parameter design of specific structures and improving the vibration resistance and isolation performance of the overall structures by adjusting the band gap frequency and the bandwidth, thereby helping to maintain the stable operation of the equipment and the physical and mental health of personnel. Analysis shows that it is reasonable to calculate the vibration band gap characteristics of periodic grillages by the WFEM. On the basis of meeting the basic strength requirements of the structures, the optimization method of structural vibration band gap performance through reasonable structural parameter design will also become a research hotspot in the future, assisted by various band gap optimization methods, such as structural topology optimization [43,44] and the replacement of piezoelectric elastic or piezothermoelastic composites [45,46]. The premise to achieve the goal is to accurately calculate and analyze the vibration band gap characteristics of periodic grillage structures.

In this article, the WFEM is used to calculate the band gap characteristics of typical periodic DwGs, which are verified by the fabrication model and the calculation of the finite element model. Simultaneously, influences of different material parameters and structural forms on band gap characteristics are analyzed, which will provide design and calculation support for the application of periodic DwGs.

## 2. Materials and Methods

**Figure 1:**
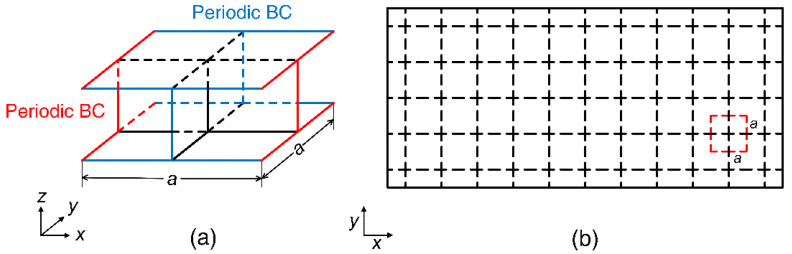
Numerical models: (**a**) unit cell for band gap calculation, where red and blue lines represent Bloch periodic boundary conditions along x- and y-axes, and (**b**) finite-length periodic structures for transfer function calculation.

As shown in Figure 1, the unit cell of a 2D periodic DwG is composed of frames in the middle and plates on top and at the bottom, which separately correspond to the sizes of 100 × 40 × 2 mm and 100 × 100 × 2 mm. The grillages are made of structural steel: density $\rho =7850\;\mathrm{kg}/m^{3}$, elastic modulus E=210 GPa, and Poisson’s ratio υ=0.28. To calculate the band gap characteristics of infinite DwGs, the periodic boundary conditions of a grillage unit cell should be determined according to Bloch theorem so that elastic wave analysis can be transformed into an eigenvalue problem.

On the basis of finite element discretization and assembly, the motion equation of a unit cell can be obtained:

(1) $$(K-\omega^{2}M)d=F$$

where ω is the circular frequency; K and M represent the overall stiffness and mass matrices of the unit cell, respectively; and d and F represent the combination of node displacement and force vectors on the left and right of, at the bottom and top of, and inside the unit cell:

(2) $$\begin{array}{c} d={\left[\begin{array}{ccccc} d_{L} & d_{R} & d_{B} & d_{T} & d_{I} \end{array}\right]}^{T}\\ F={\left[\begin{array}{ccccc} F_{L} & F_{R} & F_{B} & F_{T} & F_{I} \end{array}\right]}^{T} \end{array}$$

The force on the internal nodes of a unit cell is 0 based on the force balance condition, so the degrees of freedom of Equation (1) can be reduced:

(3) $$(K_{r}-\omega^{2}M_{r})d_{r}=F_{r}$$

where $d_{r}$ and $F_{r}$ represent node displacement and force vectors after reduction:

(4) $$\begin{array}{c} d_{r}={\left[\begin{array}{cccc} d_{L} & d_{R} & d_{B} & d_{T} \end{array}\right]}^{T}\\ F_{r}={\left[\begin{array}{cccc} F_{L} & F_{R} & F_{B} & F_{T} \end{array}\right]}^{T} \end{array}$$

According to periodic Bloch theorem, the relationship between displacement and force boundary is as follows:

(5) $$\begin{array}{c} d_{R}=e^{-iq_{x}a}d_{L},\;d_{T}=e^{-iq_{y}a}d_{B}\\ F_{R}=-e^{-iq_{x}a}F_{L},\;F_{T}=-e^{-iq_{y}a}F_{B} \end{array}$$

where $q_{k}(k=x,y)$ is the wave vector component corresponding to the first irreducible Brillouin zone boundary of a unit cell and a is the length of the unit cell in this direction.

Substituting Equation (5) into Equation (3), one can finally obtain the motion governing equation of a periodic unit cell:

(6) $$(K_{q}-\omega^{2}M_{q})d_{r}=0$$

where $K_{q}$ and $M_{q}$ represent structural stiffness and mass matrices containing wave vector $q_{k}$, respectively.

**Figure 2:**
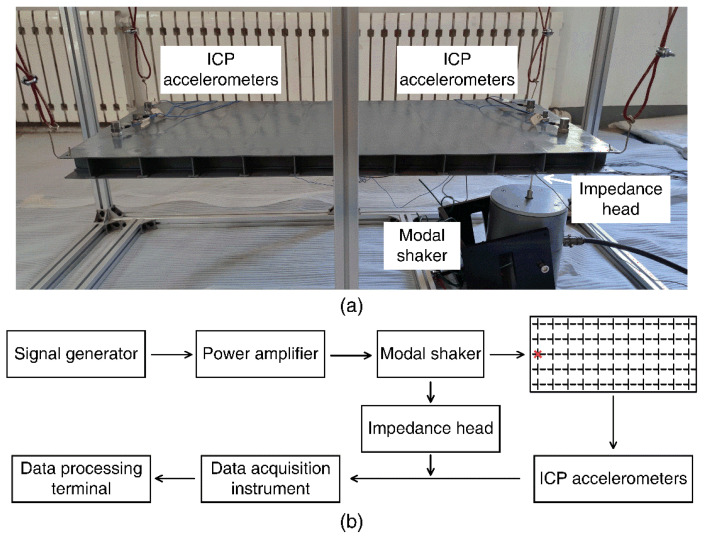
Vibration measurement (**a**) model for experiment and (**b**) process in diagram.

For the sake of validation of band gap characteristics calculated by the WFEM, the manufactured model vibration measurement experiment is carried out. As shown in Figure 2a, the finite-length test model contains 11 × 5 DwG unit cells, manufactured by argon protected welding technology, and all welds must be fully welded. To manufacture DwGs on a small scale, a layer of limited-width panel is added to the stiffeners of the SwG so that square plates can be directly welded onto the stiffener panels, which will be used as the bottom plate of the DwG.

To verify the vibration transmission characteristics of DwGs, the test model is suspended in air by wire ropes to simulate the free boundary condition and a simple harmonic excitation force of 0.02–4 kHz with intervals of 10 Hz is applied to the bottom plate at one end. Then the acceleration responses at corresponding measurement points and the excitation force at the location of modal shaker are collected to calculate the transfer function. Figure 2b shows the experiment measurement process. The excitation signal produced by the signal generator (YE1311, Sinocera, Yangzhou, China) is transmitted to the modal shaker (JZK-50, Sinocera, Yangzhou, China) through a power amplifier (YE5874A, Sinocera, Yangzhou, China), and then the data measured by ICP accelerometers (352C33*2, 353B31*4, PCB, Buffalo, NY, USA) and impedance head (CL-YD-331, Sinocera, Yangzhou, China) are transmitted to the data processing terminal through a data acquisition instrument (INV3065N2, Coinv, Beijing, China). By applying the vertical harmonic excitation at the right end of structures, the acceleration responses of measurement points at both ends can be collected so as to calculate the transfer function of finite-length periodic structures for comparative analysis according to Equation (7):

(7) $$\mathrm{FRF}=20\log(\frac{a_{o}}{a_{i}})$$

where $a_{i}$ represents the average acceleration response amplitude of the input end and $a_{o}$ represents the average acceleration response amplitude of the outlet end.

## 3. Results and Discussion

### 3.1. Numerical Calculation and Experimental Verification

**Figure 3:**
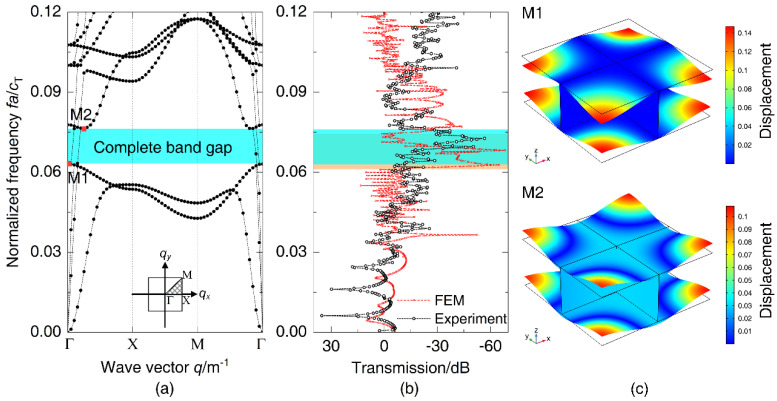
(**a**) Dispersion relations obtained in the path of the first irreducible Brillouin zone boundary, (**b**) vibration transmission spectra along the x-axis of manufactured and numerical models, and (**c**) wave propagation modes corresponding to M1–M2 in dispersion relations.

Since the plate of the manufactured model is limited to a thickness of 2.3 mm, the numerical model is re-established in light of the thickness of this section. The dispersion relations of periodic DwGs can be calculated by COMSOL Multiphysics along the path Γ(0,0)-X(1,0)-M(1,1)-Γ(0,0) that the wave vector proceeds. The path represents the boundary of the first irreducible Brillouin zone of a unit cell [33], as shown in Figure 3a. The ordinate in the graph is the normalized frequency $fa/c_{T}$, where a is the unit length and $c_{T}$ represents the transverse wave speed in steel. Figure 3b displays the transfer functions of the manufactured and finite element models in the normalized frequency range of 0–0.12, which is used to verify the vibration transmission characteristics along the x-axis. To accurately indicate the attenuation characteristics of vibration along the x-axis, the plane wave load is applied at one end of the finite element model to calculate its transfer functions.

It can be seen that periodic DwGs have a complete band gap in 0–0.12 ranging from 0.063 to 0.076, which corresponds to the vibration attenuation domain in the transmission spectra of the manufactured and finite element models, as plotted in Figure 3a,b. Within the band gap of DwGs, the vibration transmission attenuation of the test model marked in the blue zone reaches more than 56 dB. However, there is a deviation between the model test result and the band gap calculation result of the finite element model. The main reason for the deviation is that the manufacturing process of small-size double-wall grillage structures leads to a difference between the test model and the ideal finite element model. Due to the damping effect of the structures, the transfer function of the experimental model has some attenuation in the high-frequency band. To illustrate the transmission characteristics of periodic DwGs more intuitively, parts of wave propagation modes under different wave vector conditions are plotted in Figure 3c, where M1 and M2 represent modes of initial and terminal frequencies of the band gap, respectively. Both M1 and M2 manifest as shear deformation of top and bottom plates, and furthermore, the vibration is mainly concentrated in the center of the plates, which is closely related to the formation of the complete band gap. Through band gap calculation and experimental verification, it is demonstrated that the complete band gap in periodic DwGs possesses great practical application value, so the analysis of the parameter influence will focus on the band gap.

### 3.2. Analysis of Influencing Factors

**Figure 4:**
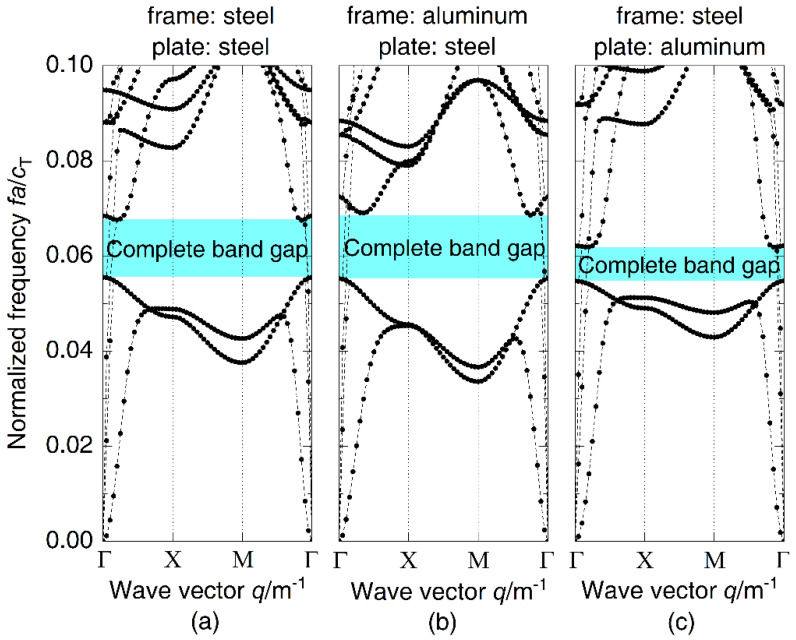
Dispersion relations of periodic (**a**) grillages with steel frames and plates, (**b**) grillages with aluminum frames and steel plates, and (**c**) grillages with steel frames and aluminum plates.

**Figure 5:**
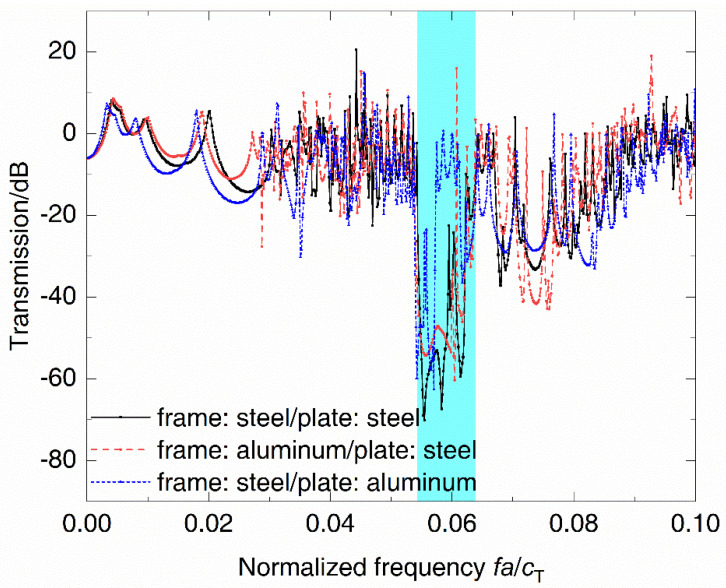
Vibration transmission spectra of periodic DwGs with components of different materials.

Based on the model in Figure 1, the material of the frames and the plates is individually substituted with aluminum to analyze the influence of material in different components on the complete band gap. The vibration band gap characteristics of all three models are shown in Figure 4, in which the original grillages with steel frames and plates are used as a reference and the material parameters of aluminum are as follows: density $\rho =2700\;\mathrm{kg}/m^{3}$, elastic modulus E=68.5 GPa, and Poisson’s ratio υ=0.34. Results indicate that the initial frequencies of the three band gaps are basically unchanged and there is some difference in terminal frequencies, which are reduced from 0.068 of grillages with steel frames and plates to 0.062 of grillages with steel frames and aluminum plates, leading to the bandwidth reduction from 0.012 of the original model to 0.007. It is obvious that the material change of plates has more influence on the complete band gap. In Figure 5, the transfer functions calculated by three numerical models of finite-length periodic structures show that the grillages with steel frames and aluminum plates have the narrowest bandwidth, in which the vibration attenuation effect is not as good as that in the other two models.

**Figure 6:**
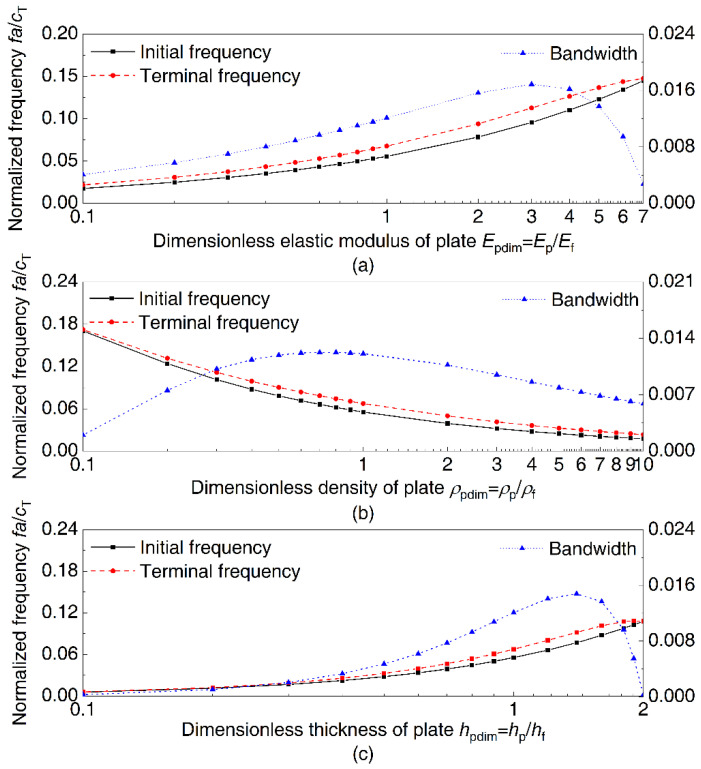
Influences of plate material properties on the complete band gap: (**a**) dimensionless elastic modulus, (**b**) dimensionless density, and (**c**) dimensionless thickness.

Since the plate has a more significant effect on the band gap, the material parameters of plates are changed to analyze the influence of elastic modulus, density, and thickness. Figure 6a shows the influence of the elastic modulus of plates on the vibration band gap of periodic DwGs. The frames are made of steel, which is used as the reference material, and the dimensionless elastic modulus of plates increases from 0.1 to 7, with other parameters being consistent with steel. The initial and terminal frequencies of the complete band gap gradually increase as the elastic modulus of the plates increases, while the bandwidth first increases and then decreases, reaching the maximum when the elastic modulus ratio equals 3. It means that the band gap will move to a higher frequency as the elastic modulus of the plates increases, but a too large or small elastic modulus will cause the bandwidth to decrease, with the attenuation effect reduced. Figure 6b displays the influence of plate density on the band gap, in which the dimensionless density of plates gradually increases from 0.1 to 10 and other parameters are consistent with steel. The frequency of the complete band gap gradually decreases as the plate density increases and the bandwidth reaches the maximum when the density ratio equals 0.7. The band gap will move to a lower frequency with an increase in the density of plates, and there is a certain density range to maximize the bandwidth. Figure 6c shows the influence of the thickness of plates on the vibration band gap of periodic DwGs. The dimensionless thickness of plates increases from 0.1 to 2, with other parameters being consistent with frames. The initial and terminal frequencies of the complete band gap gradually increase as the thickness of plates increases, and the bandwidth reaches the maximum when the thickness ratio equals 1.4.

**Figure 7:**
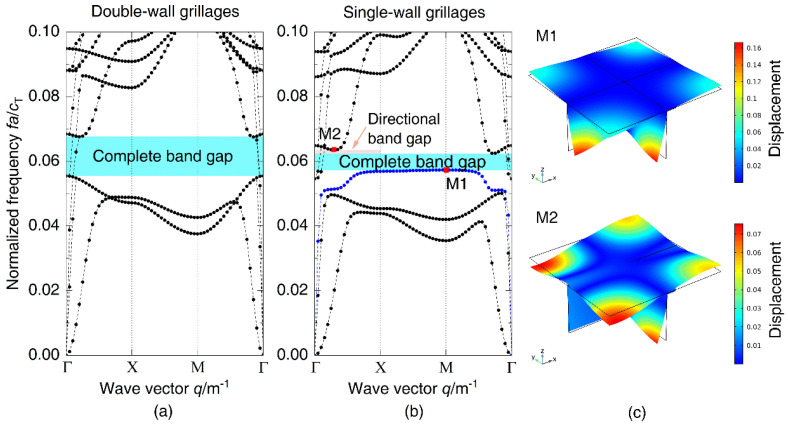
Dispersion relations of periodic (**a**) DwGs and (**b**) SwGs; (**c**) wave propagation modes corresponding to M1–M2 in dispersion relations.

**Figure 8:**
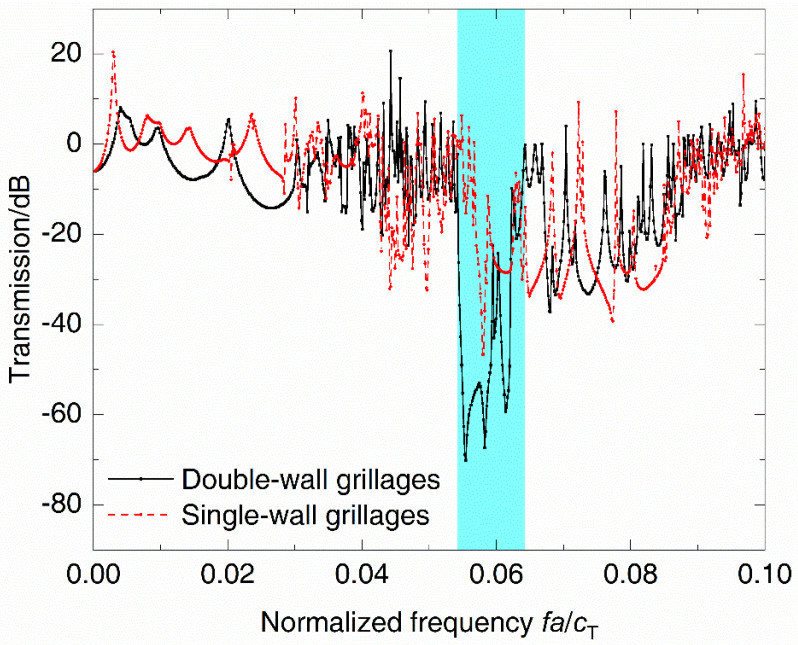
Vibration transmission spectra of periodic DwGs and SwGs.

To study the influence of plates on periodic DwGs, the band gap characteristics of DwGs and SwGs are calculated, respectively, in Figure 7a,b. Comparing the dispersion relations of the two kinds of periodic structures, it can be seen that a new, blue, dispersion curve appears in that of the SwGs and there is a new directional band gap right after the first one, which leads to a change in the frequency location and bandwidth of the band gap. In dispersion relations of SwGs, the initial frequency of the complete band gap on the new dispersion curve is selected as M1 and the terminal frequency of the directional band gap is selected as M2. The corresponding wave propagation modes are plotted in Figure 7c, where M1 displays the shear deformation of frames and M2 represents the shear deformation caused by the coupling of frames and plates. Due to the lack of restriction of plates at the bottom, both M1 and M2 exhibit shear deformation characteristics of the frames. Figure 8 shows vibration transmission characteristics of different grillage structures. Within the vibration attenuation range, the initial frequency of the complete band gap of SwGs is higher than that of DwGs and the bandwidth is narrower, as plotted in dispersion relations, which cause the attenuation effect to decrease.

**Figure 9:**
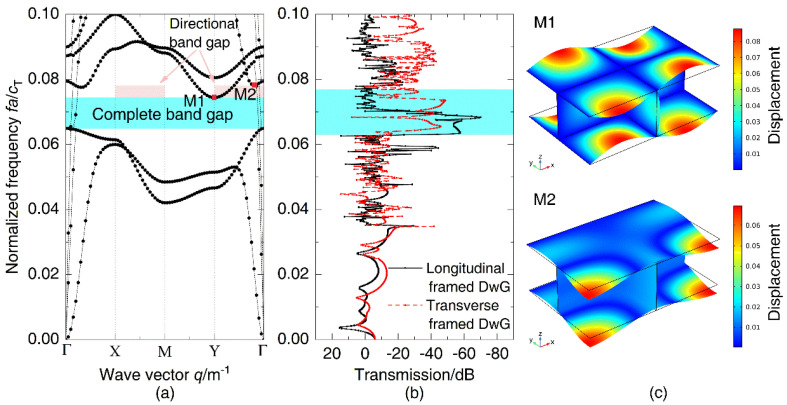
(**a**) Dispersion relations of periodic DwGs; (**b**) vibration transmission spectra of longitudinal- and transverse-framed DwGs; (**c**) wave propagation modes corresponding to M1–M2 in dispersion relations.

For engineering structures such as ships and offshore platforms, longitudinal- and transverse-framed forms of DwGs directly determine the dynamic characteristics, such as bending stiffness in the direction, which deserve more attention. This article takes the periodic DwG unit cell shown in Figure 9c as an example to analyze its dispersion relations along x- and y-axes, which correspond to the elastic wave transmission characteristics of longitudinal- and transverse-framed forms. The size of the plates is 120 × 80 × 2 mm; and the sizes of the longitudinal and transverse members in the frames are 120 × 40 × 2 mm and 80 × 40 × 2 mm, respectively. The boundary of the first irreducible Brillouin zone of the unit cell turns into Γ(0,0)-X(1,0)-M(1,1)-Y(0,1)-Γ(0,0), and the dispersion relations of DwGs can be obtained as shown in Figure 9a. The grillages contain a complete band gap and a directional band gap along the y-axis within the frequency range marked in the figure, which correspond to 0.065–0.074 and 0.074–0.078, respectively. In Figure 9c, M1 and M2 represent wave propagation modes at terminal frequencies of the complete and directional band gaps, where M1 embodies flexual wave propagation along the x-axis and M2 is formed by the coupling of shear deformation and flexual wave propagation along the y-axis. Figure 9b shows the transmission characteristics of the elastic wave in finite-length longitudinal- and transverse-framed DwGs. Within the vibration attenuation range, the transverse-framed DwGs have a wider band gap, while the longitudinal-framed DwGs have a larger attenuation amplitude.

## 4. Conclusions

In this article, the vibration band gap characteristics of 2D periodic DwGs were calculated and analyzed by the WFEM, which were verified via the model vibration experiment and finite element calculation. Then, the effects of material parameters and structural forms on the complete band gap characteristics were discussed. According to the results and analysis, the following conclusions can be drawn:

- There is a complete band gap in the periodic DwGs, which can be calculated accurately by the WFEM. The dispersion relations of numerical results are compared with the vibration transmission spectra of the model test and finite element calculation, which proves the effectiveness of the method in calculating the vibration band gap of DwGs.
- The complete band gap of periodic grillage structures is mainly related to the shear deformation of plates, and therefore material parameters of the plate have more influence on the band gap than the frame, which always works as the foundation of a DwG.
- With an increase in the dimensionless elastic modulus and thickness of plates, the band gap gradually moves to a higher frequency; in contrast, when the dimensionless density of plates gradually increases, the band gap moves to a lower frequency. All the bandwidths first increase and then decrease, and reach the maximum value when the dimensionless parameters equal certain values.
- Compared with DwGs, SwGs show shear deformation characteristics of the frames due to the lack of plates at the bottom, resulting in the decrease of the bandwidth, which causes the attenuation effect to decrease.
- The frame forms of DwGs have a direct impact on the band gap characteristics. The transverse-framed DwGs in this article produce an additional directional band gap after the complete one, which leads to a wider bandwidth and a smaller attenuation in this frequency range than the longitudinal-framed DwGs.

The above research discovered that the periodic double-wall grillage structures commonly used in engineering structures have a complete band gap. Simultaneously, the causes and influencing factors of the vibration band gap were analyzed, which makes it possible to control the vibration response of the structures by adjusting the band gap position. That is, for many engineering structures, the study of the vibration band gap characteristics of periodic DwGs will be helpful in the development of vibration and noise control. Based on the known vibration band gap characteristics of the original periodic grillage structures, the generation and use of a more practical low-frequency band gap through various methods, such as topology optimization or the introduction of composite materials, may greatly improve the vibration isolation performance of the periodic structures and become a new research hotspot.

## Data Availability

The data presented in this study are available on request from the corresponding author.
